# Artificial intelligence-oriented predictive model for the risk of postpartum depression: a systematic review

**DOI:** 10.3389/fpubh.2025.1631705

**Published:** 2025-09-03

**Authors:** Jie Xia, Chen Chen, Xiuqin Lu, Tengfei Zhang, Tingting Wang, Qingling Wang, Qianqian Zhou

**Affiliations:** School of Nursing and Health Management, Shanghai University of Medicine and Health Sciences, Shanghai, China

**Keywords:** artificial intelligence, machine learning, postpartum depression, risk, predictive model

## Abstract

**Introduction:**

Postpartum depression (PPD) is a significant mental health concern affecting 3.5-33.0% of mothers worldwide, with potentially severe consequences for both maternal and infant well-being. The emergence of artificial intelligence (AI) and machine learning (ML) technologies offers new opportunities for the early prediction of PPD risk, potentially enabling timely interventions to mitigate adverse outcomes.

**Methods:**

This systematic review was conducted until October 31, 2024, using several electronic databases, including PubMed, Web of Science, CBM, VIP, CNKI, and Wanfang Data. All the studies predicted the occurrence of PPD using algorithms. The review process involved dual-independent screening by two authors using predefined criteria, with discrepancies resolved through consensus discussion involving a third investigator, and assessed the quality of the included models using the prediction model risk of bias assessment tool (PROBAST). Inter-rater agreement was quantified using Cohen’s *κ*.

**Results:**

Eleven studies were included in the systematic review. The random forest, support vector machine, and logistic regression algorithms demonstrated high predictive performance (AUROC > 0.9). The main predictors of PPD were maternal age, pregnancy stress and adverse emotions, history of mental disorders, maternal education, marital relationship, and sleep status. The overall performance of the prediction model was excellent. However, the generalizability of the model was limited, and there was a certain risk of bias. Issues such as data quality, algorithm interpretability, and the cross-cultural and cross-population applicability of the model need to be addressed.

**Conclusion:**

The model has the potential to predict the risk of PPD and provide support for early identification and intervention. Future research should optimize the model, improve its prediction accuracy, and test its applicability across cultures and populations to reduce the incidence of PPD and guarantee the mental health of pregnant and maternal women.

## 1 Introduction

Postpartum depression (PPD) is a serious mental health problem that develops after childbirth and is characterized by persistently low mood, loss of interest or pleasure, decreased energy, and other psychological and physical symptoms (1). The incidence of PPD varies greatly between countries with different cultures and economic statuses, and its prevalence is believed to be approximately 3.5-33.0% (2). This disease may be related to various factors such as changes in hormone levels, maternal personality characteristics, marital relationships, economic status, living environment, delivery mode, and newborn health status (3). Problems related to PPD, such as tension between mothers and their newborns, difficulties in breastfeeding, and the slow growth and development of infants, are on the rise. In extreme cases, PPD may even induce suicidal behavior or a tendency to harm the baby, seriously threatening the safety and physical and mental health of the mother and baby (4, 5). PPD has become a focus of global public health. Therefore, early prediction and identification of PPD is particularly important (6). With the rapid development of science and technology, Artificial Intelligence (AI) has been widely used in obstetrics and gynecology in recent years (7–9). Machine Learning (ML) is at the core of the AI field. By building models and algorithms, the machine can continuously learn from data, self-optimize, and improve the accuracy of prediction and decision-making, and shows great potential in the early prediction and recognition of PPD (10, 11). The motivation for conducting this research is to address the critical need for early identification and intervention of PPD. While AI-ML approaches have shown promise in managing other mental health conditions, their application in PPD remains underexplored (12). This review aims to bridge this gap by providing an overview of how AI-ML can be effectively utilized in PPD management. It also aims to systematically evaluate the AI-guided PPD risk prediction model, analyze and discuss its predictive performance, applicability, and bias risk, provide a basis for the construction and optimization of future PPD risk prediction models, and provide an effective reference for early prediction, recognition, and intervention of PPD in clinical practice.

## 2 Methods

We adopted the Preferred Reporting Items for Systematic Review and Meta-Analysis (PRISMA) statement for this systematic review (13).

### 2.1 Search strategy

Databases: PubMed, Web of Science, China Biomedical Literature Database (CBM), VIP, CNKI, Wanfang Data Knowledge Service Platform; Retrieval time: October 31, 2024. Search terms included artificial intelligence (AI), machine learning (ML), algorithm, postpartum depression (PPD), maternity blues, depression after delivery, postpartum depression, postpartum, depression, postnatal depression, post-partum depression, postnatal depression, predict, prediction, risk, risk prediction, risk score, risk assessment, model, prediction model, predictive model, prognostic model, hazard, danger, and threat. The search strategy was peer-reviewed by a medical librarian using the PRESS checklist to ensure completeness. PRISMA guidelines for a systematic review were followed. The search strategy included databases such as PubMed and Web of Science, and the search string is: (“postpartum depression” or “PPD”) AND (“machine learning” or “AI” or “predictive model”).

The study protocol has not been prospectively registered in PROSPERO or other systematic review registration platforms.

### 2.2 Inclusion and exclusion criteria

The inclusion criteria were as follows: (1) the purpose of the study was to predict and diagnose patients with PPD early, (2) use of at least one ML algorithm, (3) statistical validation of the results to evaluate the performance of the model, and (4) follow-up time of <1 year postpartum.

The exclusion criteria were as follows: (1) conference abstract, literature review, and class experiment; (2) full text not available; and (3) patients who had previously been diagnosed with PPD or had undergone PPD-related medical interventions; (4) participant level data could not be used for >20% of the variables ML performance metrics were not reported (e.g., no AUROC/ACC/F1).

### 2.3 Data extraction and management

Two authors reviewed the titles, descriptions, and full texts of the collected studies based on set guidelines for inclusion and exclusion. In cases of disagreement, they decided after discussion, and a third researcher decided whether to include or exclude literature that could not be ruled on. Data extraction included publication time, research objects, research types, and main research results.

The Prediction Model Risk of Bias Assessment Tool (PROBAST) was used to evaluate the risk of bias and the applicability of the included studies. Two independent reviewers conducted an evaluation, and any disagreements were resolved through discussion. If necessary, a third reviewer made a decision. We evaluated the reliability of internal evaluators and found high consistency (Cohen’s Kappa = 0.85).

### 2.4 Data synthesis

The studies were merged using narrative synthesis. We reviewed the performance of the algorithm and the risk factors affecting PPD and evaluated the risk of bias in all included studies.

### 2.5 Grey literature sources

To minimize publication bias and capture region-specific evidence, we also searched the China National Knowledge Infrastructure (CNKI) Dissertations and Theses database, a major source of grey literature in Chinese. Master’s theses and doctoral dissertations were included given their frequent coverage of locally validated AI models in PPD screening.

## 3 Results

### 3.1 Literature search

**Figure 1:**
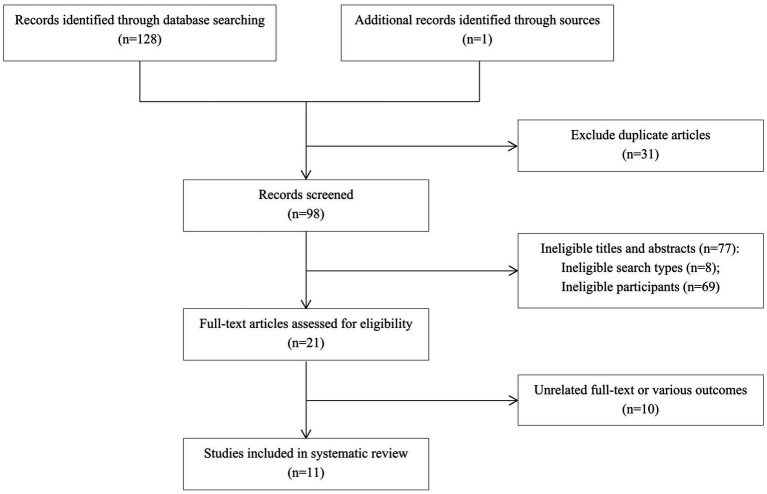
Flow diagram of the literature search and study selection.

A total of 129 articles were retrieved, including 16 from CNKI, 4 from the Wanfang Data Knowledge Service Platform, 1 from VIP Journal, 3 from the China Biomedical Literature Database, 67 from PubMed, 37 from Web of Science, and 1 from other sources. Finally, 11 articles were included (14–24) (Figure 1).

### 3.2 Articles characteristics

The articles’ basic characteristics are presented in Table 1. The studies included in this review were published between 2003 and 2023 in China (14–18), Japan (19), Israel (20), the United Kingdom (21), the United (22), and Spain (23, 24), and the sample sizes ranged from 732 to 266,544. Clear inclusion criteria were set in all 11 articles, clear exclusion criteria were set in 10 articles, and exclusion criteria were not specified in one article (15). The evaluation time points varied among the different studies, including 1 week postpartum (23), 4 weeks postpartum (19), 6 weeks postpartum (14), 4–6 weeks postpartum (17), 42 days postpartum (18), 32 weeks postpartum (24), and 1 year postpartum (20, 21). Other studies (15, 16, 22) did not indicate time points. The outcome was determined by various criteria, mainly based on Edinburgh Postnatal Depression Scale (EPDS) (14, 16–19, 23, 24), in which EPDS≥9 or EPDS≥10 were used as the threshold for determining postnatal depression. Other studies used the ICD-9/10 codes (20), PHQ-9 scale (15), PHQ-2 scale (22), and antidepressant use or non-pharmacological treatment (21) as criteria. Diagnostic interviews (DIGS) were also been used in other studies (23, 24).

**Table 1:** Basic characteristics of included studies.

Study	Country	Participants	Number of participants	Inclusion /exclusion criteria	Measurement time	Outcome indicator
Zhong et al. (14)	China	Women in Shanghai First Maternity and Infant Hospital from July 2022 to January 2023	835	Y/Y	6 weeks postpartum	EPDS≥10
Wang et al. (15)	China	Eligible pregnant women in the special research database of clinical diagnosis and treatment in Suzhou by 2023	5,814	Y/N	Unspecified	PHQ-9 ≥ 10
Deng (16)	China	A pregnant woman in an ethnic minority agricultural county in Yunnan Province in May 2022	732	Y/Y	Unspecified	EPDS≥9
Fang (17)	China	Women who gave birth in three tertiary class-A maternity and child specialist hospital in Guangzhou from August 2017 to July 2018 and met the inclusion criteria	2,396	Y/Y	4–6 weeks postpartum	EPDS≥10
Liu et al. (18)	China	Mothers who underwent cesarean section at Hunan Maternal and Child Health Hospital and Xiangya Third Hospital of Central South University from August 2014 to June 2020	1,436	Y/Y	42 days postpartum	EPDS≥10
Matsuo et al. (19)	Japan	Women who delivered at ≥35 weeks from 2014 to 2018	10,013	Y/Y	4 weeks postpartum	EPDS≥9
Hochman et al. (20)	Israel	CHS members who delivered a single child successfully and with complete information from 2008 to 2015	214,359	Y/Y	1 year postpartum	ICD-9/10 code; Diagnosed with depression; And being prescribed antidepressants
Amit et al. (21)	Britain	First-time mothers aged 18 to 45 who gave birth between 2000 and 2017	266,544	Y/Y	1 year postpartum	Diagnosed with depression; Or being treated with antidepressants; Or non-pharmacological treatments for depression
Shin et al. (22)	U. S.	Data from the US Centers for Disease Control and Prevention’s Maternal Risk Assessment Surveillance System, 2012–2013	28,755	Y/Y	Unspecified	PHQ-2 At least 1 positive answer
Jiménez-Serrano et al. (23)	Spain	Postpartum women from seven Spanish hospitals	1,397	Y/Y	1 week postpartum	If EPDS≥9, the evaluation was performed using DIGS
Tortajada et al. (24)	Spain	White women who gave birth at seven Spanish general hospitals between December 2003 and October 2004	1,397	Y/Y	32 weeks postpartum	EPDS≥9 were evaluated using the Spanish version of DIGS

### 3.3 AI-oriented ML algorithms and their performance

The best ML algorithms and their performance indicators for the included studies are listed in Table 2. The frequency and AUROC of each ML algorithm in the included studies are listed in Table 3.

**Table 2:** The best ML algorithms and their performance indicators for the included studies.

Study	Machine learning algorithms	Best algorithm	AUROC	ACC	PRE	REC/SEN	SPE	F1	G	Brier score
Zhong et al. (14)	LR, SVM, RF	RF	Training set: 0.925 Test set: 0.943	Training set: 0.871 Test set: 0.903	Training set: 0.860 Test set: 0.684	Training set: 0.553 Test set: 0.722	-	Training set: 0.670 Test set: 0.703	-	Training set: 0.091 Test set: 0.073
Wang et al. (15)	LR, RF, SVM, XGBoost, BP	RF	Prenatal: 0.849 Postpartum: 0.864	-	-	Prenatal: 0.844 Postpartum: 0.891	Prenatal: 0.854 Postpartum: 0.836	-	-	-
Deng (16)	RF1 (7 variables), RF2 (12 variables), RF3 (19 variables)	RF1	0.873	0.873	0.727	0.258	0.983	0.381	0.504	-
Fang (17)	BN, NB, Decision Tree J48, RF, ANN, SVM, LR	BN	0.763	0.717	0.714	0.717	0.799	0.714	-	-
Liu et al. (18)	LR, SVM, RF, XGBoost, LGBM, MLP	XGBoost	Training set: 0.789 Test set: 0.744	Training set: 0.702 Test set: 0.676	-	Training set: 0.767 Test set: 0.773	Training set: 0.686 Test set: 0.656	Training set: 0.5 Test set: 0.45		Training set: 0.129 Test set: 0.132
Matsuo et al. (19)	LR, Ridge Regression, Elastic Net, Kernel-based SVM, RF	Elastic Net	0.701 (Model 3)	-	-	-	-	-	-	-
Hochman et al. (20)	XGBoost	XGBoost	0.712	-	-	0.349	0.905	-	-	-
Amit et al. (21)	GBM	GBM	0.844	-	-	0.764	0.80	-	-	-
Shin et al. (22)	kNN, RPART, SVM, GBM, RF, NNET, NB, LR, AdaBoost	RF	0.884	0.791	0.839	0.732	0.865	0.776	-	-
Jiménez-Serrano et al. (23)	NB, LR, SVM, ANN	NB	0.75	0.73	-	0.72	0.73	-	0.73	-
Tortajada et al. (24)	ANN	ANN	0.84	0.84	-	0.78	0.85	-	0.81	-

-, Not mentioned.

**Table 3:** The frequency and AUROC of each ML algorithm in the included studies.

Machine learning algorithm	Frequency of use	AUROC_min_	AUROC_max_	0.7 < AUROC ≤0.8	0.8 < AUROC ≤0.9	AUROC > 0.9
RF	14	0.613	0.943	2	7	2
SVM	12	0.530	0.925	5	1	2
LR	11	0.626	0.926	5	1	2
ANN	6	0.66	0.84	0	4	0
NB	3	0.75	0.793	3	0	0
XGBoost	5	0.712	0.800	4	1	0
Ridge Regression	3	0.630	0.702	1	0	0
Elastic Net	3	0.628	0.701	1	0	0
GBM	2	0.712	0.844	1	1	0
BP	2	0.771	0.782	2	0	0
LGBM	2	0.727	0.771	2	0	0
MLP	2	0.706	0.757	2	0	0
BN	1	-	0.763	1	0	0
kNN	1	-	0.776	1	0	0
RPART	1	-	0.789	1	0	0
NNET	1	-	0.704	1	0	0
AdaBoost	1	-	0.857	0	1	0
Decision Tree J48	1	-	0.656	0	0	0

-, Not mentioned.

The Random Forest (RF) algorithm has demonstrated excellent performance in multiple studies. Zhong et al. (14) showed that the AUROC values of the RF algorithm in the training and test set 6 weeks after delivery reached 0.925 and 0.943, respectively, and the accuracy rates reached 0.871 and 0.903, respectively. Brier scores were 0.091 and 0.073, respectively, which were lower than those reported by Liu et al. (18). In addition, the RF algorithm was used the most frequently among all algorithms, reaching 14 times, and the AUROC value exceeded 0.9 two times, indicating its stability and high accuracy in predicting PPD and that the RF algorithm can handle large-scale data sets and evaluate the importance of each feature.

Support Vector Machine (SVM) and Logistic Regression (LR) algorithms are commonly used, which have been used 12 times and 11 times, respectively. The maximum AUROC value of the SVM algorithm in predicting depression risk at 6 weeks postpartum was 0.925 (14), which showed high efficiency in dealing with nonlinear problems. The LR algorithm has been used in several studies (14, 15, 17, 18, 22, 23). Compared to other algorithms, the LR algorithm is simple, easy to understand, and has strong interpretability. However, this requires a highly linear assumption for the data, and the prediction results may be biased when there is a nonlinear relationship in the data.

The Artificial Neural Network (ANN) algorithm, as the best algorithm in Tortajada et al. (23), has an AUROC value of 0.84 and an accuracy rate of 0.84, showing its ability to process complex data patterns. However, ANN is rarely used, possibly because of its high requirements for data volume and feature engineering. To train ANN models with good performance, a large amount of high-quality data is usually required as support. However, the acquisition and processing of medical data are often limited by ethics, privacy, resources, and other aspects, which are difficult to achieve. On the other hand, feature engineering is one of the key steps in the construction of ANN model, which involves extracting useful features from the original data to better describe and predict the target variables. However, this process is often complicated and time-consuming, and the medical explanation of the influence of the respective variables on the dependent variables in the model remains unclear.

The Extreme Gradient Boost (XGBoost) algorithm was used five times, and the AUROC value exceeded 0.8 one time (15). In a study by Liu et al. (18), the AUROC value of the training set was 0.789 and its accuracy was 0.702, indicating high specificity and sensitivity. It was also listed as the best algorithm by Hochman et al. (20), with an AUROC value of 0.712, demonstrating its advantage in handling unbalanced datasets. However, many model parameters and meticulous parameter adjustments are required to achieve the best performance.

Other algorithms such as Gradient Boosting Machines (GBM), Bayesian networks (BN), Decision Tree (DT), etc. have also been applied in various studies. However, they are used less frequently, possibly because they are not as stable as RF, SVM, and LR on specific datasets.

The observed difference in AUROC values across different outcome measures could be due to the inherent properties of each tool. The EPDS is specifically designed to assess postpartum depression and has been widely validated, which might contribute to its higher sensitivity and specificity. In contrast, PHQ-2/9 and DIGS, while also valid tools, might not be as specifically tailored to the postpartum period, potentially affecting their performance in this context. Further research is needed to explore the impact of outcome measure selection on the performance of AI-based predictive models for PPD.

Additionally, we conducted a thorough assessment of the model optimizations and validations used in each study to understand their impact on model performance (Table 4). This included hyperparameter settings, hyperparameter selections and feature selections. Furthermore, we analyzed the strategies employed to address class imbalance, such as propensity score matching (PSM), synthetic minority oversampling technique (SMOTE), and the use of class weights, as these techniques can significantly influence the model’s ability to generalize from the training data to unseen data. Model validations were rigorously carried out using methods including 10-Fold CV, random split validation, and temporal validation, ensuring a reliable assessment of model performance.

**Table 4:** Model optimizations and validations of the best algorithms in included studies.

Study	Best algorithm	Model optimization			Model validation	
		Hyperparameter setting/selection	Feature selection	Imbalanced handler	Internal	External
Zhong et al. (14)	RF	Grid search	SFS	-	10-Fold CV	Temporal validation
Wang et al. (15)	RF	-	Univariate analysis Multicollinearity removal	PSM	5-Fold CV	-
Deng (16)	RF1	mtry = 2, ntree = 250	SWSFS	-	Random split validation	-
Fang (17)	BN	Grid search	-	-	10-Fold CV	-
Liu et al. (18)	XGBoost	Grid search		PSM	10-Fold CV	-
Matsuo et al. (19)	Elastic Net	-	-	SMOTE	Fold CV Derivation dataset	Validation dataset
Hochman et al. (20)	XGBoost	-	SHAP	-	Random split validation	Temporal validation
Amit et al. (21)	GBM	-	SHAP	-	Random split validation (pooled 3-Fold CV)	Geographical validation Temporal validation Holdout test
Shin et al. (22)	RF	-	Relief	-	10-Fold CV	-
Jiménez-Serrano et al. (23)	NB	-	Pruning	-	Random split validation	-
Tortajada et al. (24)	ANN	-	Pruning	-	Random split validation	-

-, Not mentioned.

### 3.4 AI-oriented predictors of PPD

**Figure 2:**
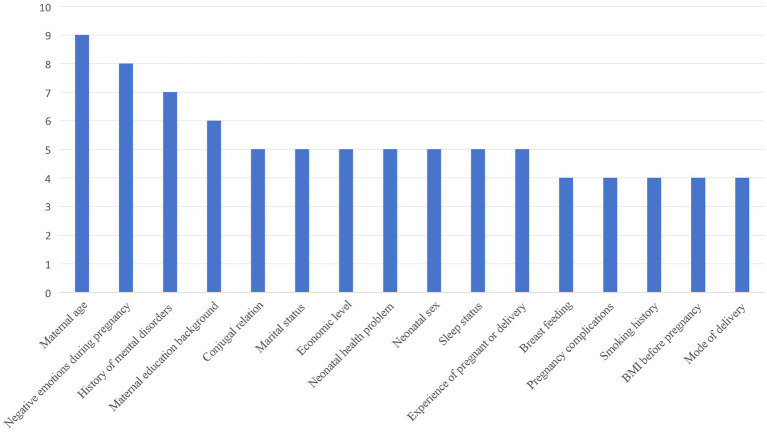
Predictors of PPD mentioned more than 3 times in included studies.

As shown in Table 5, various social demographic, psychological, obstetric, and clinical-related factors were associated with the occurrence of PPD. More than one-third of the included studies mentioned the same predictors of PPD (>3 times), and the frequency of these factors suggested a high correlation in predicting PPD (Figure 2).

**Table 5:** Predictors of PPD in included studies.

Study	Predictors of PPD		
	Sociodemographic factors	Psychology and Social psychological factors	Obstetrical and clinical related factors
Zhong et al. (14)	Age ≤25, marital status, high school education or below, return to work 6 weeks after delivery	Postpartum 3d depression, postpartum anxiety at 6 weeks, postpartum 3d worries about newborn health, postpartum relationship with in-laws at 6 weeks, postpartum relationship with husband at 6 weeks, domestic violence experience	Neonatal admission to observation room, breastfeeding at 6 weeks postpartum, sleep quality at 6 weeks postpartum, history of poor pregnancy, monocyte count
Wang et al. (15)	Whether the only child, the impulse to lose temper to the child	EPQ, SSRS, TCSQ, GAD	PSQI
Deng (16)	Age, place of residence, age of spouse	In the last 1 year, experienced negative events, marital feelings, financial worries about having a child, gender expectations of the pregnant woman for the child, gender expectations of the family for the child, previous history of negative emotions, anxiety during pregnancy, and level of social support	Previous pregnancy history, previous delivery history, number of pregnancies, self-rated health status of pregnant women, evaluation of fetal health status of pregnant women, sleep status, time to exercise each time, bad living habits
Fang (17)	Maternal age, spouse age, maternal income, spouse income, maternal education level, spouse education level, whether the only child, confinement place after discharge	Relationship with the husband, relationship with the husband’s parents (mother-in-law and daughter-in-law), relationship with their parents, whether the pregnancy is planned, the sex of the baby when pregnant, stressful events in life, confidence in raising the baby, and whether the medical care is worried	Time of delivery, method of delivery, sleep status, feeding method of the present baby, breastfeeding experience
Liu et al. (18)	-	EPDS score during pregnancy, stress level during pregnancy, domestic violence, emotional changes during pregnancy, relationship between mother-in-law and daughter-in-law, and propensity to self-harm during pregnancy	-
Matsuo et al. (19)	Age, years of education, age of husband, marital status, sex of newborn	Husband’s presence at the time of delivery, mental state, and history of mental disorders	First delivery, history of infertility treatment, number of induced abortions, hypertensive disorders during pregnancy, placental previa or placental hypoposition, placental abruption, mode of delivery, induction of labor, blood loss during delivery, postpartum transfer, height (newborn), birth weight, small for gestational age, 1 min and 5 min Apgar scores, umbilical artery blood pH, congenital abnormalities, pre-pregnancy weight, pre-pregnancy BMI, height, husband’s smoking status during pregnancy, sleep status, duration of sleep before pregnancy, breastfeeding status, smoking history
Hochman et al. (20)	Maternal age, marital status, country of birth (immigrant or not), ethnicity (Arab, Jewish), socioeconomic status, baby sex	Chronic mental illness prior to pregnancy	Preconception BMI, smoking status during pregnancy, Chalson Comorbidity Index (CCI), preconception chronic physical disease, previous number of live births, Adjusted Clinical Group (ACG) score during pregnancy, laboratory data during pregnancy, pregnancy complications, gestational weeks during delivery, birth weight, preterm birth, overdue pregnancy, mode of delivery, birth complications, congenital malformation
Amit et al. (21)	Age, race, deprivation index	Previous history of depression and anxiety	Total drug prescriptions before and during pregnancy, past antidepressant prescriptions, number of diagnoses, number of any lab tests, abdominal pain, beta-blocker prescriptions, premenstrual syndrome, smoking, pre-pregnancy BMI
Shin et al. (22)	Age, race/ethnicity, education level, marital status, income, baby sex	Stress during pregnancy, depressive symptoms during pregnancy	Pregnancy complications, preterm birth, small for gestational age, weeks of breastfeeding, pre-pregnancy BMI, alcohol consumption during pregnancy, changes in smoking behavior during pregnancy, postpartum physical recovery, pre-pregnancy oral hygiene, and exercise during pregnancy
Jiménez-Serrano et al. (23)	Age, education level, employment during pregnancy, family income level, sex of the baby, number of family members living with the mother	Personal and family history of psychosis, mood changes during pregnancy, life events after delivery, neurotic and depressive symptoms	-
Tortajada et al. (24)	Age, education level, marital status, employment during pregnancy, family income level, sex of the baby, number of family members living with the mother	Personal and family history of psychosis, mood changes during pregnancy, number of life events after delivery, neurotic and depressive symptoms, and level of social support	Combination of 5-HTT-GC genotypes, medical problems during pregnancy, drug use during pregnancy (including alcohol and tobacco), medical problems with more days in hospital, medical problems with newborns, cesarean section, use of anesthesia during delivery, medical problems with the mother during delivery

-, Not mentioned.

#### 3.4.1 Socio-demographic factors

Maternal age, educational level, marital status, family income level, sex of the baby, socioeconomic status, and whether the mother was an only child were mentioned in multiple studies. Maternal age is considered an important predictor and has been widely discussed in several studies. Some studies have shown that mothers aged less than 25 years are at a high risk of PPD (14).

#### 3.4.2 Psychological and socio-psychological factors

Chronic mental illness before pregnancy, stress during pregnancy, depressive symptoms during pregnancy, personal and family histories of mental illness, mood changes during pregnancy, life events after delivery, neurotic and depressive symptoms, level of social support, and Eysenck’s personality test results are important predictors (15, 19–24).

#### 3.4.3 Obstetrical and clinical-related factors

These included pre-pregnancy BMI, smoking status during pregnancy, alcohol consumption during pregnancy, exercise during pregnancy, mode of delivery, gestational week during delivery, birth weight, preterm delivery, overdue pregnancy, delivery complications, neonatal health problems, breastfeeding status, pregnancy complications, and medical problems before and during pregnancy (14–24).

### 3.5 Assessment of risk of bias

As shown in Table 6, all studies demonstrated a risk of bias, and the main reason for the bias was the utilization of a retrospective design. Specifically, among the predictors, the risk of bias of the predictors in Shin et al. (22)‘s study was not clear, and the predictors in the other studies were all low-risk. Some studies (15, 16, 22) did not specify the outcome evaluation time; therefore, there was a high risk of bias. In the analysis items, some studies (14–20, 22–24) had problems, such as the lack of universality of the selected research objects or insufficient sample size. For example, Deng (16) selected pregnant women from agricultural counties inhabited by ethnic minorities in Yunnan Province, whereas Tortajada et al. (24) selected white women. In addition, Amit et al. (21) did not report scores on the PPD-related scale, so there was a high risk of bias in the overall study. Among the applicability evaluations, eight studies were rated as high risk (14–18, 20, 23, 24) and three as low risk (20–22).

**Table 6:** Results of bias and applicability risk assessment according to PROBAST.

Study	Risk of bias			Applicability			Overall		
	Participants	Predictors	Outcome	Analysis	Participants	Predictors	Outcome	Risk of bias	Applicability
Zhong et al. (14)	+	−	−	+	+	−	−	+	+
Wang et al. (15)	+	−	+	+	+	−	−	+	+
Deng (16)	+	−	+	+	+	+	−	+	+
Fang (17)	+	−	−	+	+	−	−	+	+
Liu et al. (18)	+	−	+	+	+	−	−	+	+
Matsuo et al. (19)	+	−	−	?	−	−	−	+	−
Hochman et al. (20)	+	−	−	+	+	−	−	+	+
Amit et al. (21)	+	−	−	+	−	−	−	+	−
Shin et al. (22)	+	?	+	+	−	−	−	+	−
Jiménez-Serrano et al. (23)	+	−	−	+	+	−	−	+	+
Tortajada et al. (24)	+	−	−	+	+	+	−	+	+

+, High risk of bias/low applicability; −, Low risk of bias/high applicability; ?, Unclear. The four items in the field of bias risk cover a total of 20 questions, each question needs to be answered with “yes,” “no,” “unclear.” When all the questions under a certain item are answered with “yes,” it indicates that the item has a low risk of bias; All items in the field of applicability are evaluated according to “good applicability,” “poor applicability” and “unclear.” If all items have good applicability, then the overall applicability of the study is good.

## 4 Discussion

### 4.1 The AI-oriented PPD risk prediction models have good performance

Research shows that the prevalence rate of PPD is approximately 10-20% in the world. China is a populous country with a large number of pregnant women. The incidence rate of PPD in China is 15–20%, and nearly 5–7 million women suffer from depression (25, 26). Traditional screening and diagnostic methods often rely on questionnaires and clinical interviews, which are subjective, time-consuming, and laborious. The introduction of AI technology, particularly the application of ML algorithms, has provided new perspectives for the early identification and prediction of PPD. These algorithms can improve prediction accuracy by building complex models and mining predictors closely related to the occurrence of PPD from massive data. The results of this study show that existing PPD models have good performance indicators. Domestic scholars (14–16) show that the AUROC value of the RF algorithm in the training and test sets reaches the highest values of 0.925 and 0.943, respectively, and the average AUROC value exceeds 0.8. The prediction results were integrated with the classification results of multiple decision trees, and the final results were more reliable (27, 28). In addition, some domestic scholars (17, 18) also used the XGBoost and BN algorithms to rapidly process high-dimensional and multi-classification tasks, and the AUROC values exceeded 0.75, showing good prediction performance (29). These results indicate that AI technology in China can effectively and quickly extract risk factors for PPD from complex medical data and provide support for clinical decision-making.

Globally, the application of AI technology for PPD risk prediction has great potential and application prospects. Researchers in different countries and regions have begun to explore the effectiveness of ML algorithms for predicting PPD risk. These studies not only provide valuable data support for local areas, but also provide possibilities for international comparison and cooperation. ML algorithms, such as the RF and GBM algorithms, have strong adaptability in predicting PPD risk. Shin et al. (22) showed that a model based on the RF algorithm had good predictive performance in American pregnant women, with an AUROC value of 0.884. Amit et al. (21) applied the GBM algorithm with an AUROC value of 0.844, and its performance was better than that of similar algorithms used in domestic studies (18) (XGBoost algorithm). This not only provides guidance for local medical practice, but also provides a reference for other countries.

Compared with other algorithms, the Random Forest (RF) algorithm demonstrated superior performance in identifying key factors influencing postpartum depression and provided quantitative analysis, which is crucial for timely and effective interventions. Its comprehensive evaluation mechanism allows it to handle complex datasets and evaluate the importance of each feature, contributing to its high accuracy and stability in predicting PPD (30). The robustness of the RF algorithm in adapting to large-scale datasets has been widely recognized (31), making it particularly suitable for the analysis of extensive medical data commonly encountered in PPD research. Furthermore, the adjustment or setting of hyperparameters in RF, such as the number of trees and their depth, plays a critical role in optimizing model performance (32, 33). These parameters can significantly influence the model’s ability to generalize from the training data to unseen data, as evidenced by the high AUROC values reported in several studies included in this review (14, 15). Therefore, it is essential to continue strengthening the application of RF in PPD prediction and to explore its full potential through meticulous hyperparameter tuning and feature selection processes.

### 4.2 AI-oriented main predictors of PPD

This study showed that maternal age was the main predictor of PPD. First-time mothers may experience self-blame and anxiety due to their lack of reproductive experience and the need to care for their newborns while recovering from the postpartum period, often experiencing small practical difficulties that are difficult to resolve quickly. These feelings can increase over time and lead to PPD (34). Stress and poor mood during pregnancy are major predictors of PPD. During pregnancy, women experience significant hormonal changes that affect emotional regulation and increase their sensitivity to stress. Furthermore, concerns about childbirth, changes in body image, and anxiety about the future role of motherhood can add to the psychological burden (35). The lack of adequate social support can exacerbate this sense of isolation, making it more difficult for pregnant women to cope with these challenges (36). This study also showed that a history of mental disorders was a predictor of PPD. Studies have speculated that PPD is mostly a continuation of prenatal psychological problems and mood disorders, and that the risk of depression increases after childbirth due to a sharp drop in hormones in the mother’s body (37). In addition, maternal education, marital relationship, sleep status, and other factors were highly correlated with PPD, which is consistent with a number of previous studies (38). It is worth noting that pre-pregnancy BMI is also an important predictor of PPD, and further studies on the mechanism of PPD should be conducted.

### 4.3 Challenges of AI-oriented PPD risk-prediction model

#### 4.3.1 Risk of bias in the studies

Although existing AI-oriented PPD risk-prediction models have good prediction performance, the overall risk of bias is high, and their applicability still needs to be strengthened. This study indicated that all included studies had a risk of bias. The research objects selected by different studies are regional, which will cause a certain selection bias, and the processing methods of missing data in each study are not unified, which may lead to confounding bias that will interfere with the research results and the prediction of predictors. Therefore, in future studies, it will be necessary to strictly refer to each item in the PROBAST evaluation method, reasonably deal with existing and missing data, design a more targeted risk-prediction model for PPD according to different populations, and select the best matching algorithm model according to different sample sizes and research purposes.

#### 4.3.2 Cross-culture and cross-population applicability of the algorithm models

China is a multiethnic and multicultural country, with significant differences in cultural customs, social and economic conditions, and medical resource distribution in different regions. Together, these factors influence the risk factors and manifestations of PPD. Studies indicate that women in rural areas face a higher risk of PPD due to issues such as neonatal sex, living conditions, and lack of medical insurance (39). In addition, different regions in China may have different assessment and diagnostic criteria for PPD, making it difficult to directly compare and apply data collected in different regions under the same model. Therefore, when developing and applying PPD risk-prediction models in China, regional differences must be considered, and the models must be adjusted and optimized accordingly to ensure their applicability across different cultures and populations.

Internationally, in addition to traditional predictors, race is an important factor leading to PPD, emphasizing the importance of local adjustment of PPD risk-prediction models in different cultural contexts (21). Fang (17) adjusted the outcome criteria according to the results of Deng et al. (40) and obtained a PPD screening index that was more suitable for Guangzhou. In addition, different studies have reported different outcomes of PPD. Studies mainly used EPDS to assess PPD in China. Studies by Jimenez-Serrano et al. (23) and Tortajada et al. (24) used the EPDS combined with the DIGS to evaluate the outcome of PPD. Therefore, a model developed in one country may not be applicable to others (41). Future research should be conducted on a global scale, summarizing commonalities and differences among scales and adjusting the weights of different impact factors in the algorithm model to verify and enhance its universality.

#### 4.3.3 Data quality and algorithm interpretability

ML algorithms, particularly deep learning models, are considered “black-box” models that lack transparency in their decision-making processes, which is an important limiting factor for clinical applications (42). In clinical practice, doctors and patients must understand the basis of a model’s predictions to better accept and apply those predictions. Ethical and privacy concerns cannot be ignored when applying AI. Patients are highly sensitive to medical information and there are differences in their acceptance of new technologies and methods (43). Therefore, in the process of popularization and application, it is necessary to fully respect the wishes and privacy of patients, formulate strict data management and privacy protection policies, strengthen doctor-patient communication, and improve patient awareness and trust in AI prediction models. Moreover, future research should use interpretable machine learning models, such as SHAP values or LIME, to improve model transparency and clinical applicability. This will help clinical doctors better understand and trust predictive results, thereby more effectively applying these models in practical clinical environments.

### 4.4 Strengths and limitations

This systematic review aimed to synthesize AI-oriented prediction models for the risk of PPD. It not only focuses on the prediction performance and applicability of AI-oriented ML algorithms but also provides valuable insights for continuous challenges and further responses.

This study has several limitations. Owing to the limitations of the retrieved database, our findings may not cover all available evidence, and we cannot explicitly claim that these are the only relevant results. We excluded studies published in languages other than Chinese or English, which may have limited the scope and comprehensiveness of our search. Moreover, we could not quantitatively analyze the predictive performance of all AI-oriented ML algorithms because the performance indicators reported in some studies were inconsistent.

Another limitation of this study is that the study protocol failed to be prospectively registered on a public registration platform (such as PROSPERO) before the start of the study. This means that the research process lacks pre declared and publicly verifiable plans, which may increase the potential risk of selective reporting bias. We acknowledge that this is an important defect of this study, and similar studies in the future will strictly comply with the norms of prospective registration.

In addition, the variability in PPD definitions poses challenges for generalizing AI models. It highlights the need for more standardized approaches to outcome assessment and model development in future research. This could involve the use of common data collection protocols, standardized assessment tools, and agreed-upon definitions of PPD (44). By addressing these issues, future studies may be better positioned to provide more consistent and generalizable findings.

## 5 Conclusion

This study reviewed 11 studies that used AI-oriented PPD risk-prediction models. We comprehensively summarized the basic characteristics, predictive performance, and main predictors of all studies and evaluated their applicability and risk of bias. We found that AI-oriented PPD risk-prediction models are still in the developmental stage. They have a good overall prediction performance, but the overall risk of bias is high, such as data quality, algorithmic interpretability, cross-cultural and cross-population applicability, and ethical and privacy challenges that still need to be overcome. In the future, efforts should be made to improve and optimize the prediction model, strengthen its verification and evaluation, regularly calibrate model performance, and continuously update the algorithm data. It is necessary to strengthen the supervision and regulation of AI technology to ensure its legitimacy and compliance with practical applications (45). Most importantly, interdisciplinary cooperation should be strengthened to jointly promote in-depth research and the wide application of AI in the prediction of PPD risk and reduce the risk and incidence of PPD in pregnant women, thereby ensuring the mental health of pregnant women and promoting the harmony of pregnant women, their families, and society.

## Data Availability

The original contributions presented in the study are included in the article/supplementary material, further inquiries can be directed to the corresponding author.
